# Supplementary material for: TRIM56-mediated monoubiquitination of cGAS for cytosolic DNA sensing
Source: Nat Commun. 2018 Feb 9;9:613. doi: 10.1038/s41467-018-02936-3 (PMC5807518; doi:10.1038/s41467-018-02936-3)
Supplement: Supplementary file 1 — Supplementary Information [file 41467_2018_2936_MOESM1_ESM.pdf]

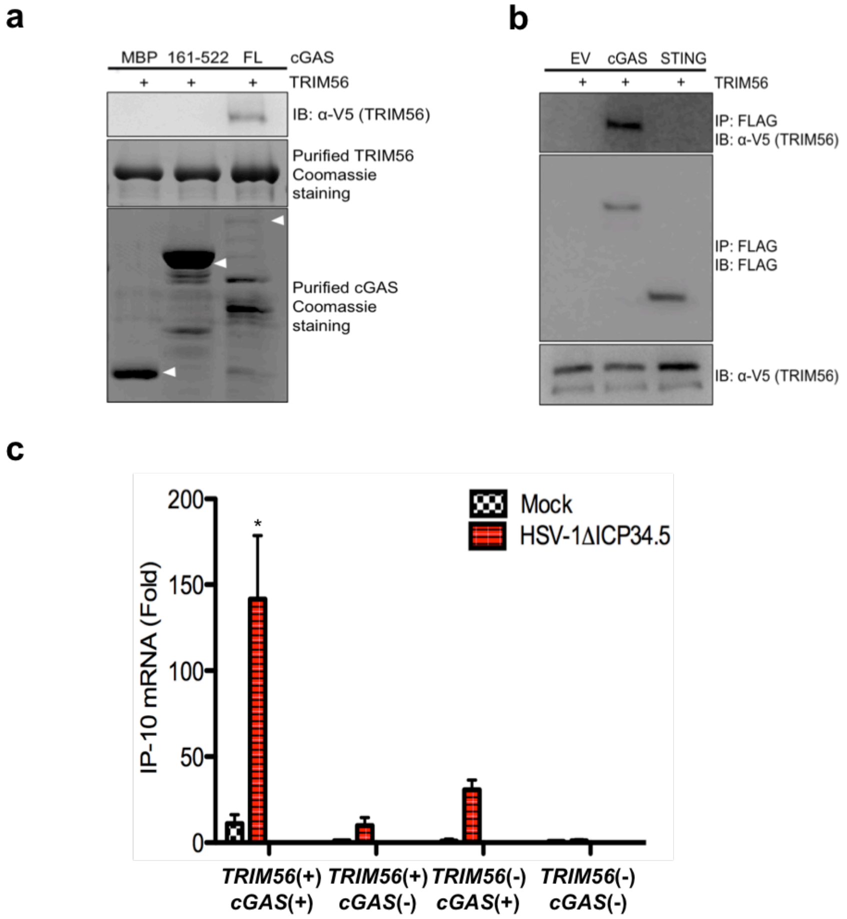

Supplementary Figure 1. TRIM56 directly binds to the cGAS N-terminus and is required for IP-10 induction. (a) Maltose-binding protein (MBP), fusion MBP-cGAS C-terminus (aa 161-522), and fusion MBP-cGAS full-length protein were purified from *E. coli*. TRIM56 was purified from 293T cells. A co-immunoprecipitation assay was conducted using amylose resin. White arrows indicate MBP (left), MBP-cGAS C-terminal 161-522aa (middle) and MBP-cGAS full-length (right), respectively. **(b)** TRIM56 binds to cGAS, but not STING. 293T cells were co-transfected with TRIM56-V5 and empty vector (EV), cGAS-FLAG, or STING-FLAG. After 48 hours, cGAS or STING co-immunoprecipitate was washed with 500mM NaCl followed by immunoblotting. **(c)** cGAS-deleted L929 cells with or without TRIM56 deletion were complemented with wild- type cGAS-FLAG (the cell lines generated for Figure 2) before infection with HSV-1ΔICP34.5 (MOI=5). The expression of IP-10 mRNA was measured using real-time PCR. Data (a-b) are representative of two independent experiments. Data (c) are representative of three independent experiments. Error bars indicate mean ± s.d of n=3 (c). *P<0.05 versus control using Student's *t*-test (c). Full blots are shown in Supplementary Fig. 10.

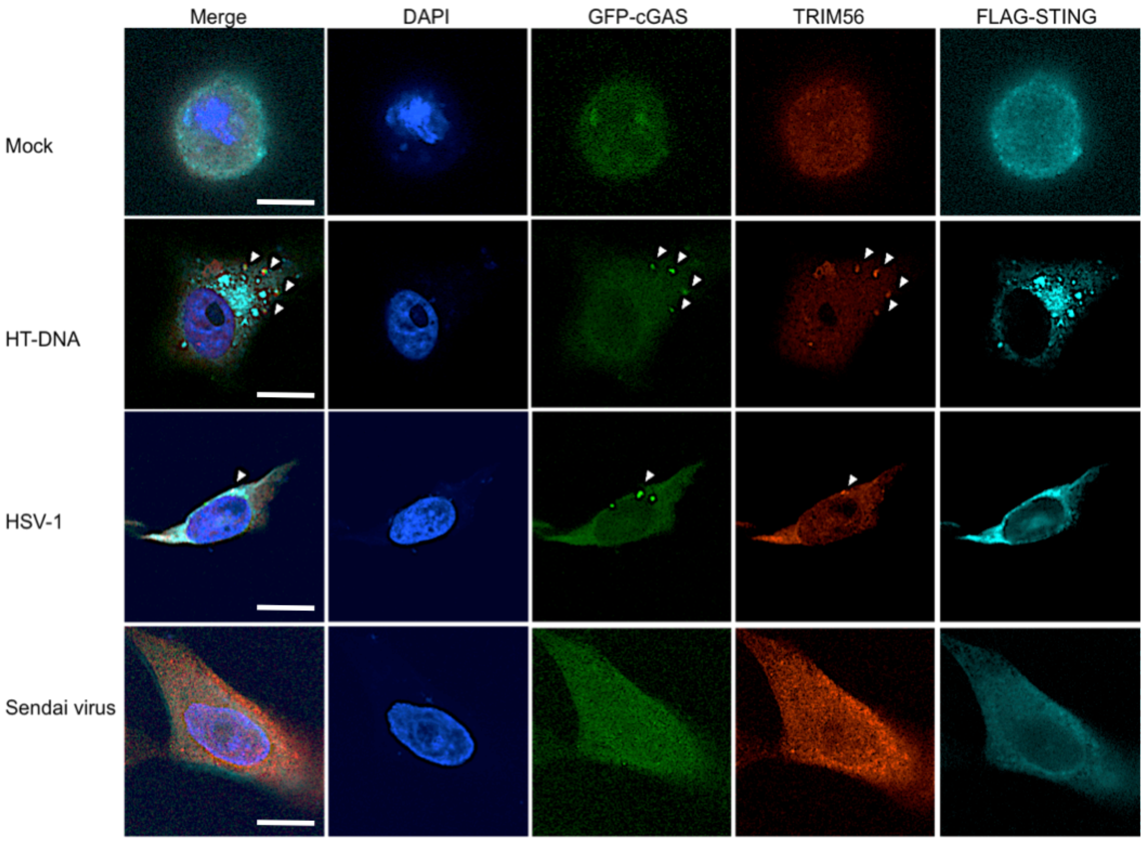

Supplementary Figure 2. TRIM56 and cGAS co-localize at foci in response to DNA stimulation and infection with DNA virus, but not RNA virus. GFP-cGAS and FLAG-STING constructs were exogenously expressed in HeLa Cells for 48 hours before stimulation with herring testis DNA (HT-DNA; 2μg/ml), infection with Herpes Simplex Virus 1 (HSV-1), or infection with Sendai virus. Co-localization of cGAS with endogenous TRIM56 was observed at the indicated foci (see arrowhead). Data are representative of three independent experiments. Scale bar, 5 μM.

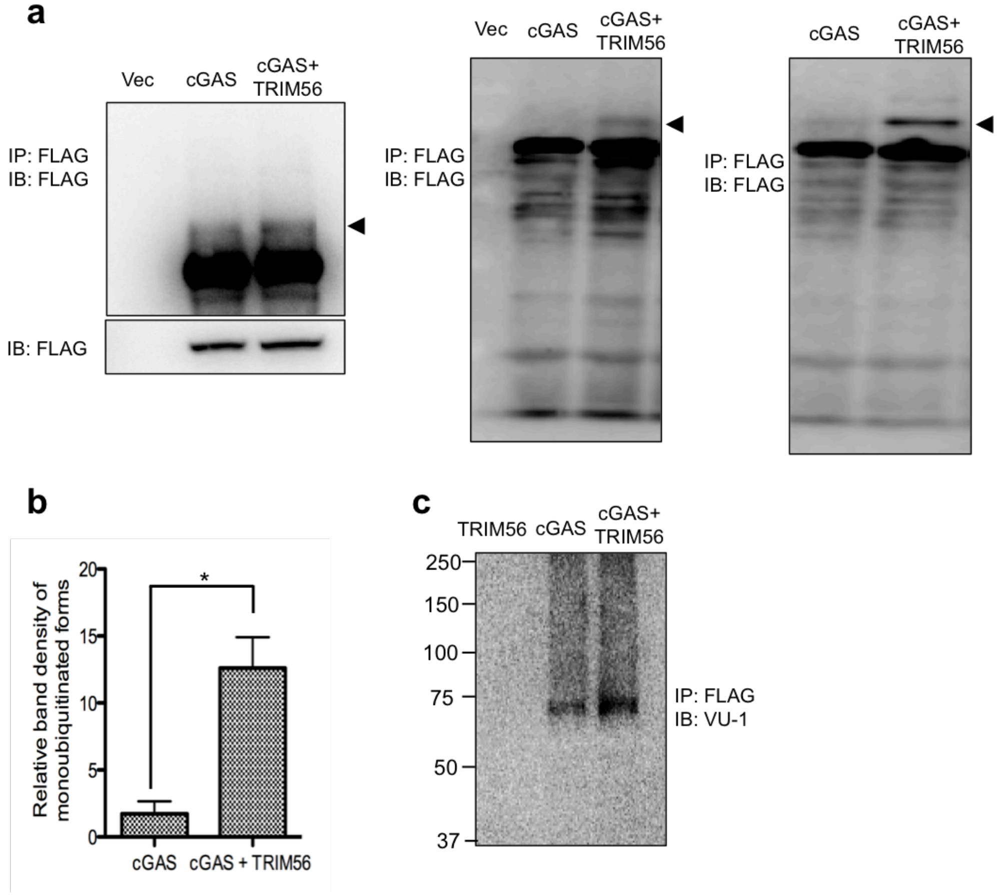

Supplementary Figure 3. TRIM56 leads to monoubiquitination of cGAS. (a) In three independent trials, cGAS complexes were pulled down from 293T cells transfected with empty vector, cGAS-FLAG alone, or cGAS-FLAG and TRIM56-V5. Western blot was performed using FLAG antibody. Band shifts (arrowhead) are indicated. **(b)** For cells overexpressing cGAS with or without TRIM56 co-transfection, the densities of the cGAS band shifts from (a) were quantitated relative to the background. **(c)** In the ubiquitination assay of Figure 3b, a single band near 75kDa was observed, suggesting preferential monoubiquitination. Data (a-b) show three independent experiments. Error bars indicate mean ± s.d of of three independent experiments (b). **P*<0.05 versus control using Student's *t*-test (b). Full blots are shown in Supplementary Fig 10.

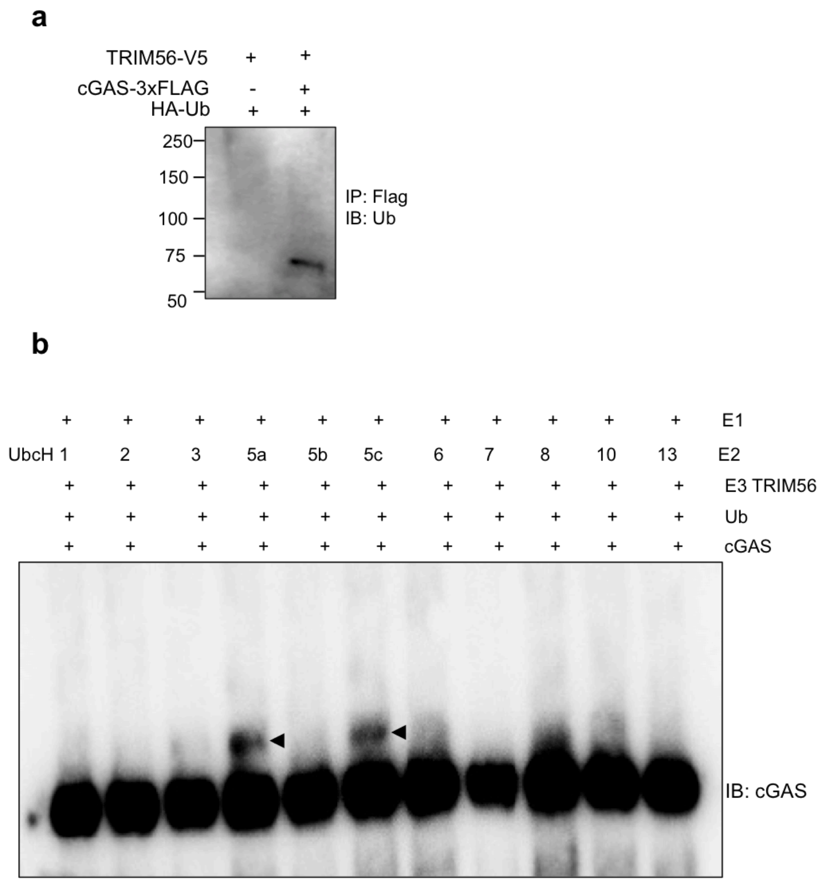

Supplementary Figure 4. TRIM56 triggers the monoubiquitination of cGAS *in vitro*. (a) HEK293T cells were transfected with TRIM56-V5, cGAS-3xFLAG or HA-Ub. 24 hours after transfection, whole cell lysates (WCLs) were used for immunoprecipitation and immunoblotting, as indicated. **(b)** An *in vitro* ubiquitination assay with the indicated combinations of a mixture of E1, E2 (UbcH; numbers above lanes indicate enzyme variants), E3 (TRIM56), cGAS and Ubiquitin (Ub). Immunoblot of the *in vitro* ubiquitination was detected by cGAS antibody. Data are representative of two independent experiments (b). Full blots are shown in Supplementary Fig. 10.

a

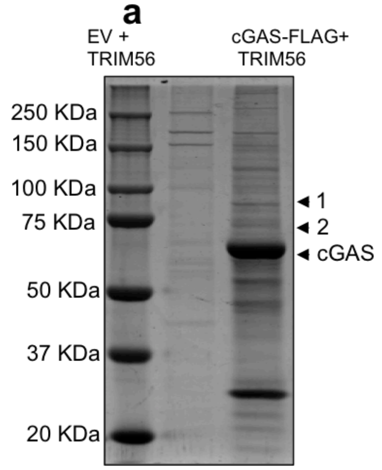

b

ScanF	z	↓ XCorr	ΔCorr	# Ions	Peptide
10036	3	1.725	0.056	14/40	R.EPFYLVPKNAK#.D
15794	3	1.44	0.331	13/52	K.EGLPIQGWLGTK#VR.T
17369	2	1.961	0.596	10/18	R.NNGFPIFDK#L.
5068	2	3.189	0.778	20/26	K.NAK#DGNSFQGETWR.L
7304	2	2.68	0.581	14/20	K.EIK#DIDVSVEK.E
8661	3	1.604	0.009	12/44	R.REPFYLVPKNAK#.D

Supplementary Figure 5. Identification of cGAS ubiquitination sites. (a) Purification and Coomassie blue stain of cGAS-FLAG from HEK293T cells transfected with TRIM56 and empty vector (EV), or with TRIM56 and cGAS-FLAG. **(b)** Summary of mass spectrometry data identifying cGAS ubiquitination sites.

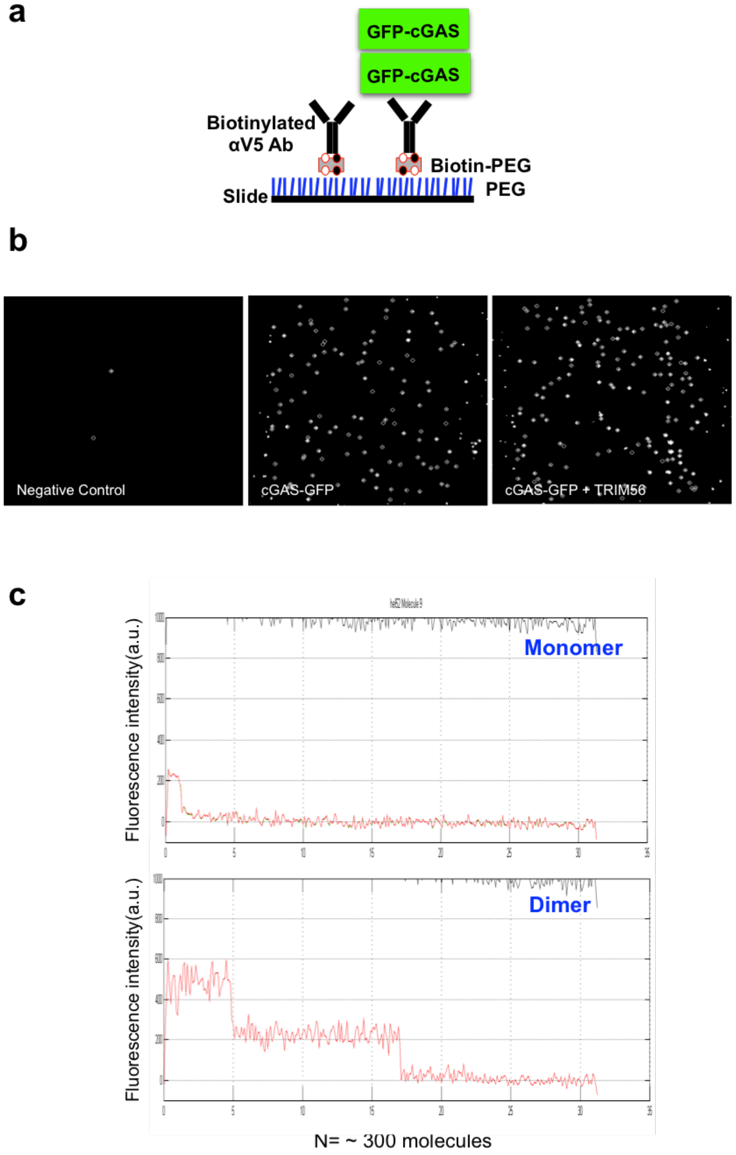

Supplementary Figure 6. GFP-cGAS photobleaching traces. (a) Schematic depiction of GFP-cGAS SiMPull. **(b)** Representative GFP fluorescence images. **(c)** Sample GFP time traces depicting one- or two-step photobleaching. For determining the stoichiometry, traces were manually scored for the number of bleaching steps. The graph shows representative photobleaching curves with one- and two-step photobleaching. >300 traces were scored to reliably identify the photobleaching step distribution.

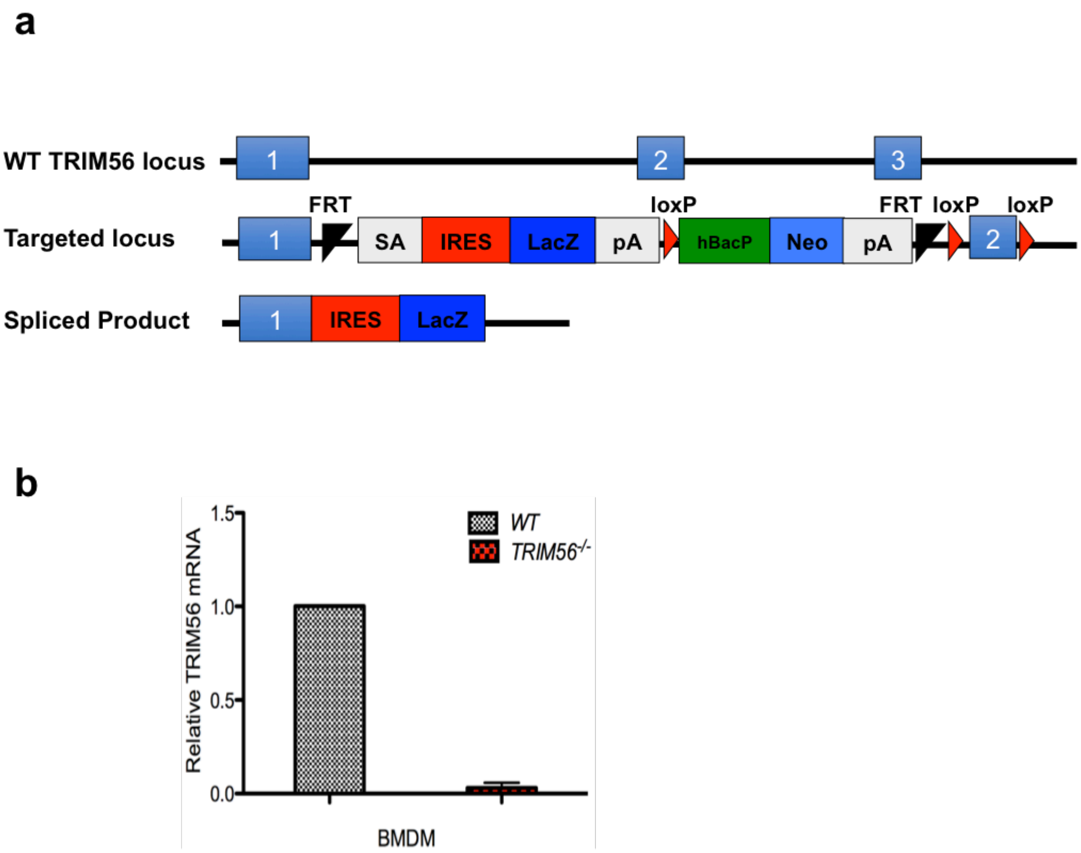

Supplementary Figure 7. Generation of *TRIM56*$^{-/-}$ mice. (a) Schematic representation of the *TRIM56 WT* locus showing its exons (boxes) and introns (lines), and the locus disrupted by the targeting vector. The splice acceptor (SA) sequence in the LacZ cassette interrupts the normal splicing of the *TRIM56* gene, resulting in the loss of *TRIM56* expression. **(b)** RT-PCR analysis of *TRIM56* mRNA amplified from the total RNA of bone marrow-derived macrophages (BMDMs) from *WT* or *TRIM56*$^{-/-}$ mice.

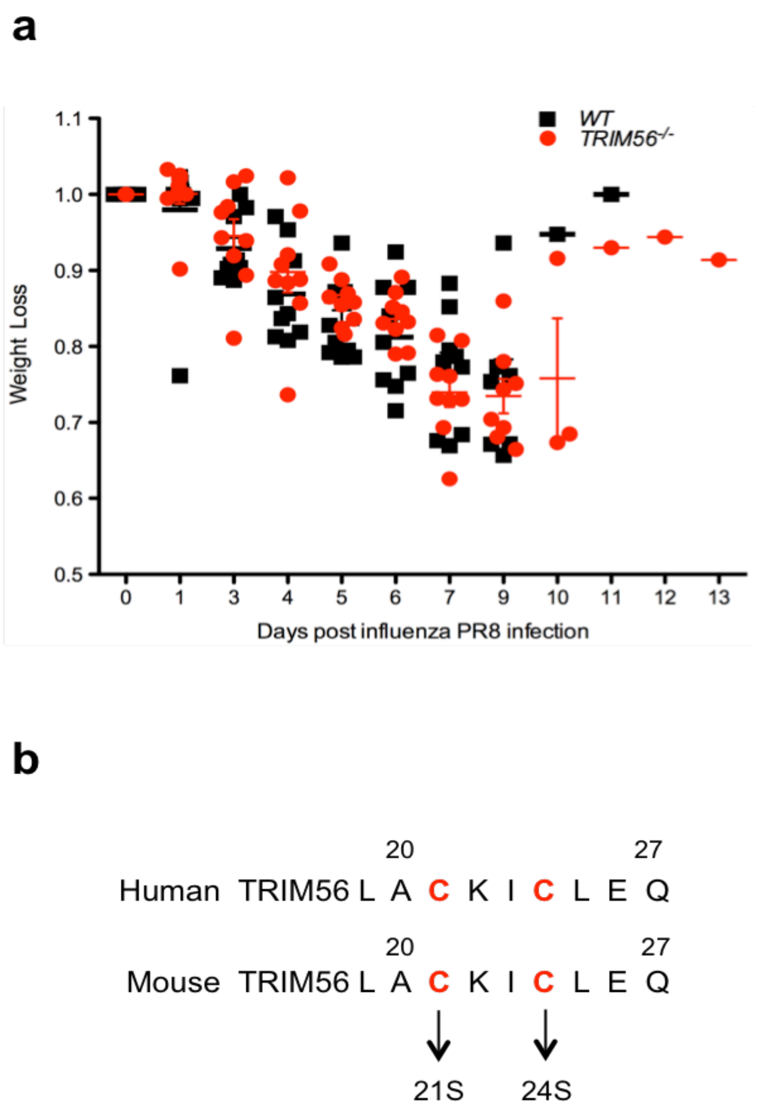

Supplementary Figure 8. Weight loss over time in both *WT* and *TRIM56*$^{-/-}$ mice after influenza PR8 infection and the mutation for TRIM56 E3 ligase-deficiency. (a) Percent body weight loss over time of both individual *WT* and *TRIM56*$^{-/-}$ mice (each n=9) infected intranasally with 1000pfu IAV PR8. **(b)** Sequence within the TRIM56 RING domain containing the conserved cysteine residues responsible for E3 ligase activity. Influenza PR8 (1000 pfu/mouse) was administered intranasally to *WT* or *TRIM56*$^{-/-}$ mice (n=9 each). Data (a) are representative of two independent experiments.

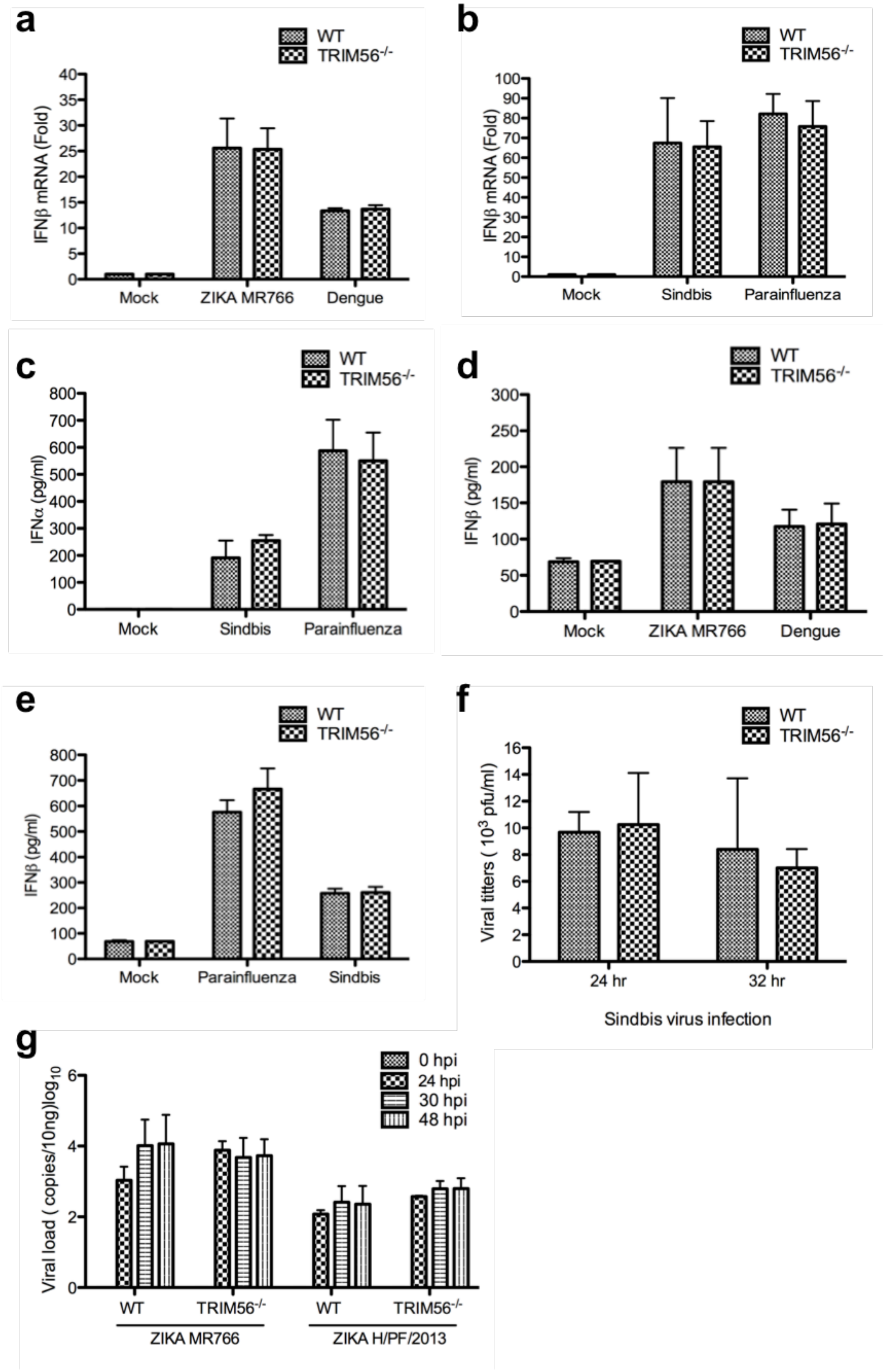

a
WT
TRIM56-/-
IFNβ mRNA (Fold)
Mock
ZIKA MR766
Dengue
b
WT
TRIM56-/-
IFNβ mRNA (Fold)
Mock
Sindbis
Parainfluenza
c
WT
TRIM56-/-
IFNα (pg/ml)
Mock
Sindbis
Parainfluenza
d
WT
TRIM56-/-
IFNβ (pg/ml)
Mock
ZIKA MR766
Dengue
e
WT
TRIM56-/-
IFNβ (pg/ml)
Mock
Parainfluenza
Sindbis
f
WT
TRIM56-/-
Viral titters (10^3 pfu/ml)
24 hr
32 hr
Sindbis virus infection
g
0 hpi
24 hpi
30 hpi
48 hpi
Viral load (copies/10ng)log10
WT
TRIM56-/-
WT
TRIM56-/-
ZIKA MR766
ZIKA H/PF/2013

Supplementary Figure 9. TRIM56 does not affect the host innate immune response to positive- and negative-sense RNA viruses. (a) Fold change in *IFNβ* expression in *WT* and *TRIM56*$^{-/-}$ BMDMs in response to ZIKV African strain MR766 and Dengue virus (MOI=5). **(b)** Fold change in *IFNβ* expression in *WT* and *TRIM56*$^{-/-}$ BMDMs in response to Sindbis virus and Parainfluenza virus (MOI=5). **(c)** Magnitude of *IFNα* secretion in *WT* and *TRIM56*$^{-/-}$ BMDMs in response to Sindbis virus and Parainfluenza virus (MOI=5). **(d)** Magnitude of *IFNβ* secretion in *WT* and *TRIM56*$^{-/-}$ BMDMs in response to ZIKV MR766 and Dengue virus (MOI=5). **(e)** Magnitude of *IFNβ* secretion in *WT* and *TRIM56*$^{-/-}$ BMDMs in response to Sindbis virus and Parainfluenza virus (MOI=5). **(f)** Viral titers of *WT* and *TRIM56*$^{-/-}$ BMDMs infected with Sindbis virus. The plaque formation assay was performed on Vero cells. **(g)** Viral load over time of *WT* and *TRIM56*$^{-/-}$ BMDMs infected with MR766 African lineage ZIKV or H/PF2013 Asian lineage ZIKV. Data (a-b, g) are representative of three independent experiments. Data (c-f) are representative of two independent experiments. Error bars indicate mean ± s.d of n=3 (a-e, g), n=4 (f).

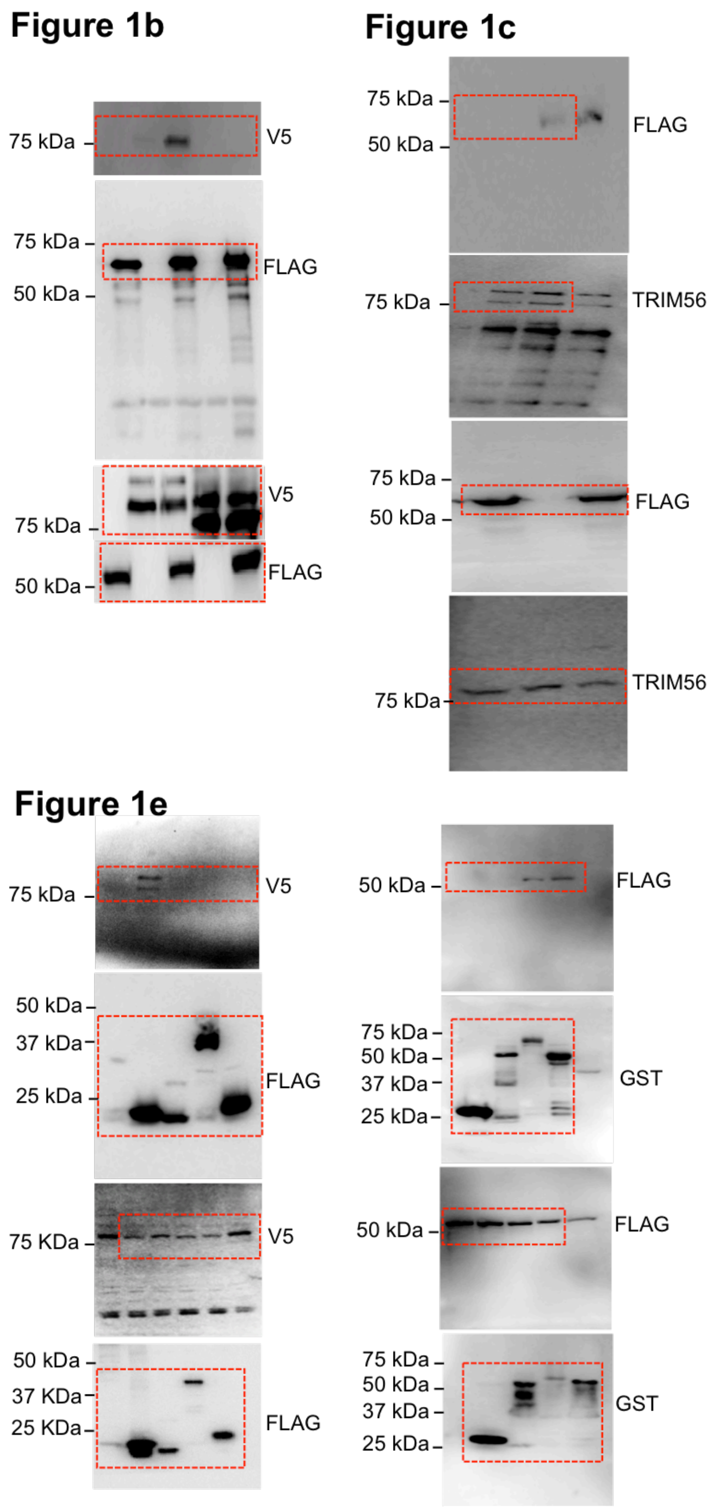

Supplementary Figure 10. Full-length uncropped western blots.

Uncropped Western blot images for Figure 1. Cropped areas are marked by red box.

Figure 2a

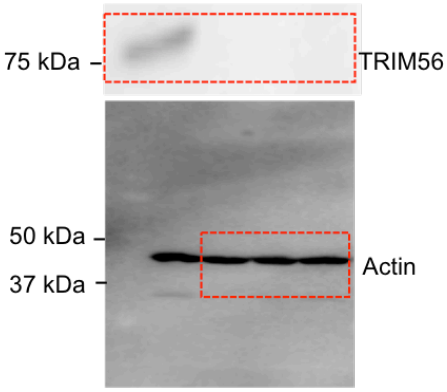

Supplementary Figure 10, cont. Full-length uncropped western blots. Uncropped Western blot images for Figure 2. Cropped areas are marked by red box.

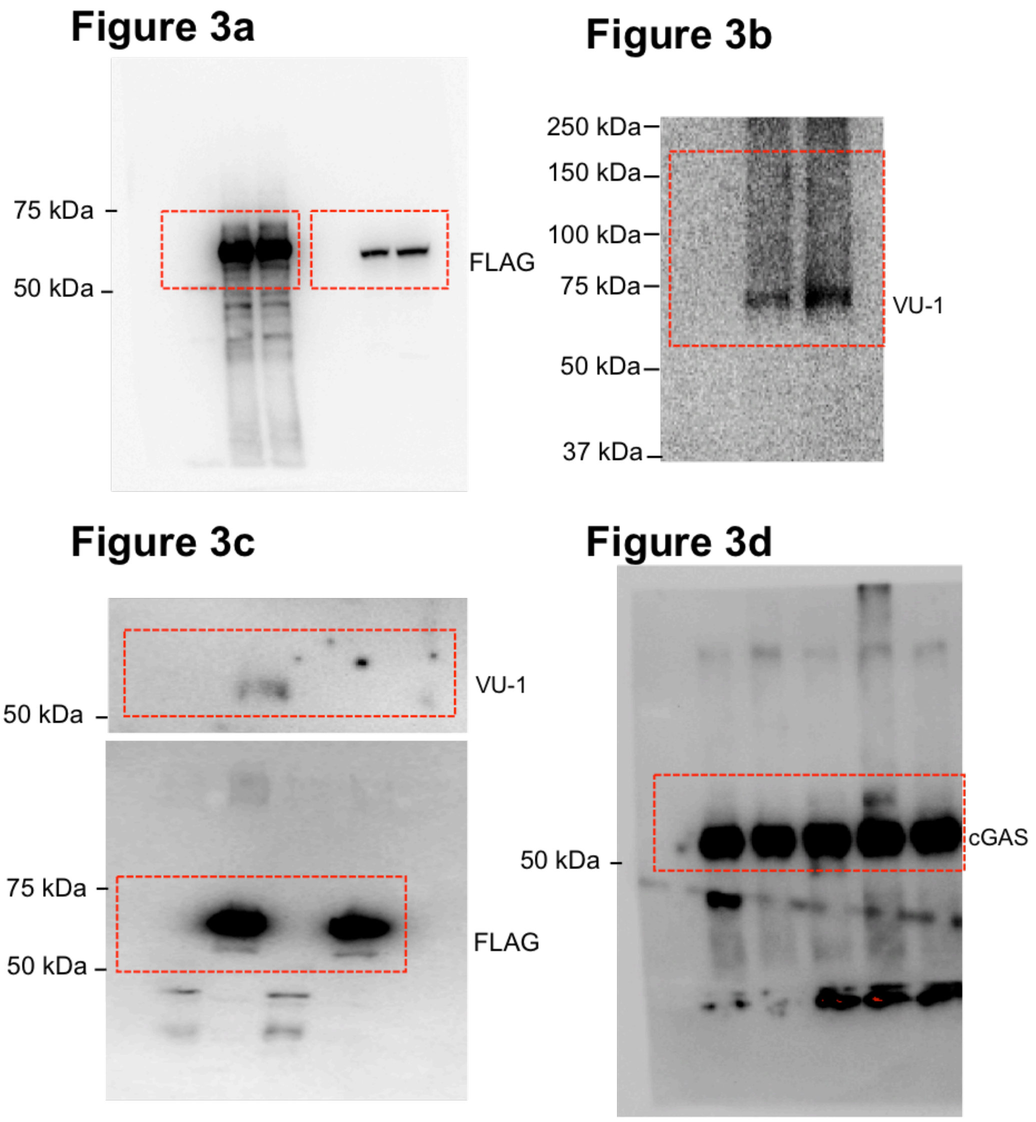

Supplementary Figure 10, cont. Full-length uncropped western blots. Uncropped Western blot images for Figure 3. Cropped areas are marked by red box.

Figure 4a

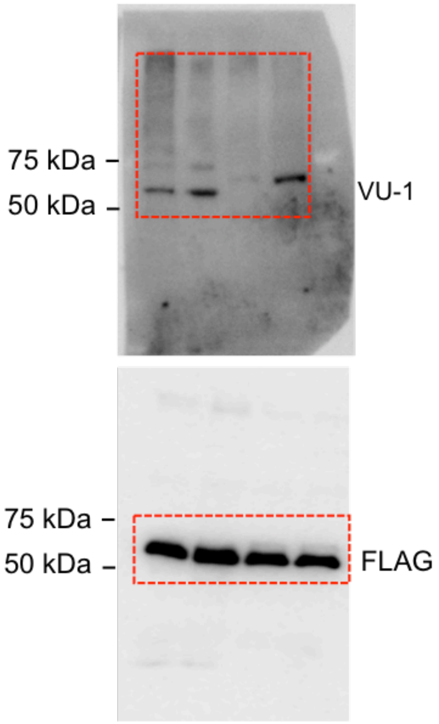

Figure 4d

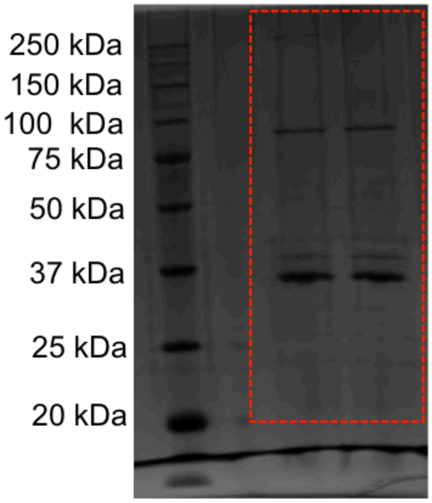

Supplementary Figure 10, cont. Full-length uncropped western blots. Uncropped Western blot images for Figure 4. Cropped areas are marked by red box.

Supplementary Figure 1a

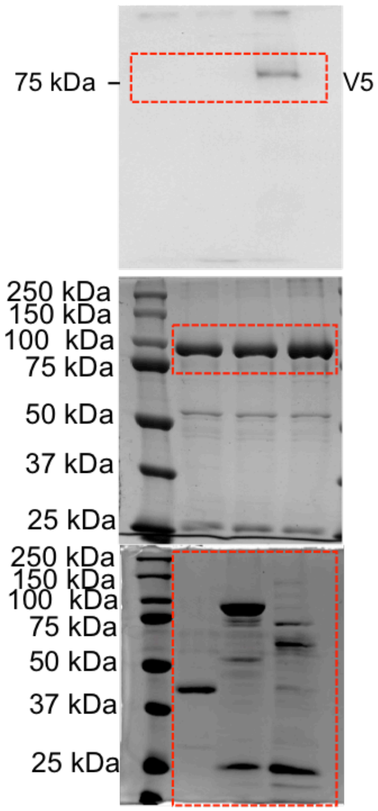

Supplementary Figure 1b

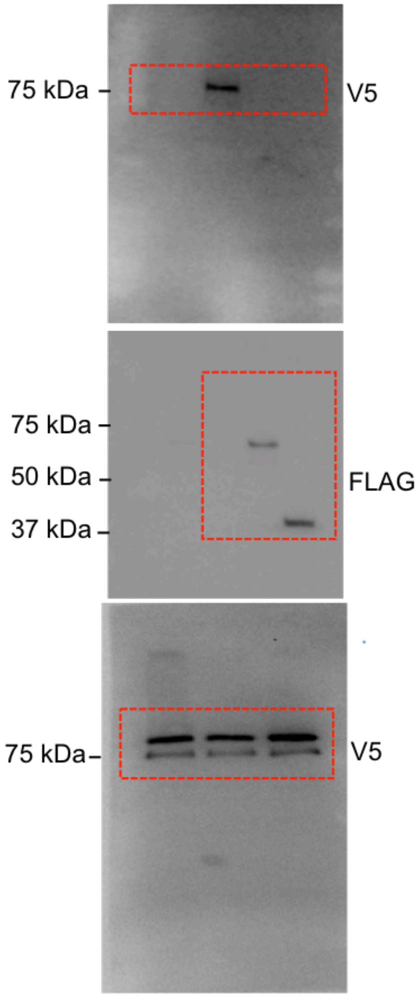

Supplementary Figure 10, cont. Full-length uncropped western blots.

Uncropped Western blot images for Supplementary Figure 1. Cropped areas are marked by red box.

Supplementary Figure 3a

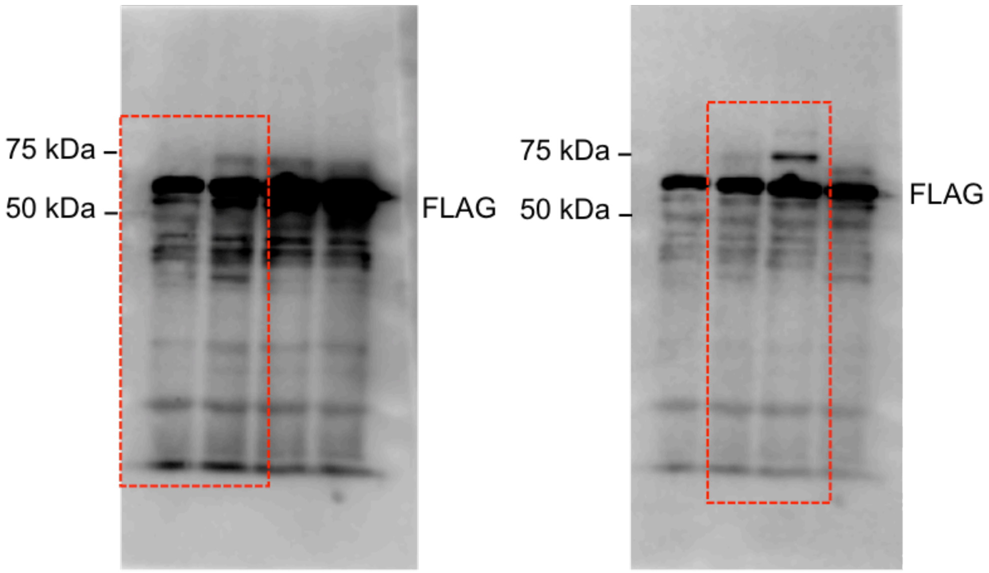

Supplementary Figure 3c

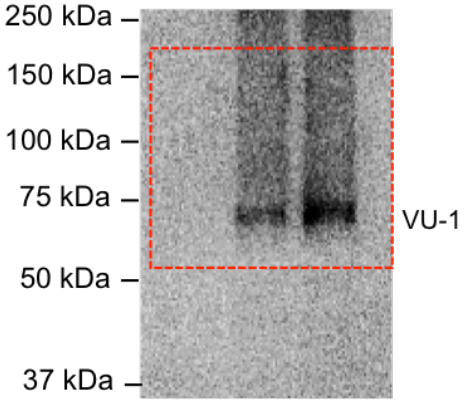

Supplementary Figure 10, cont. Full-length uncropped western blots.

Uncropped Western blot images for Supplementary Figure 3. Cropped areas are marked by red box.

Supplementary Figure 4a

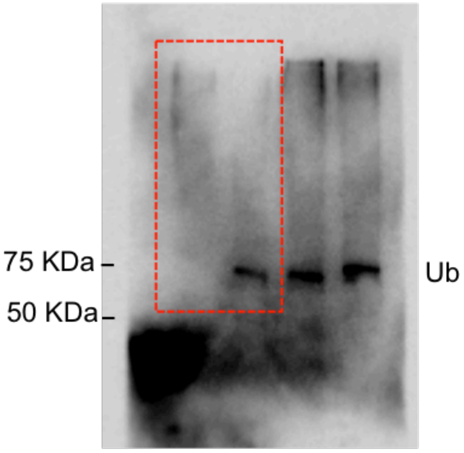

Supplementary Figure 4b

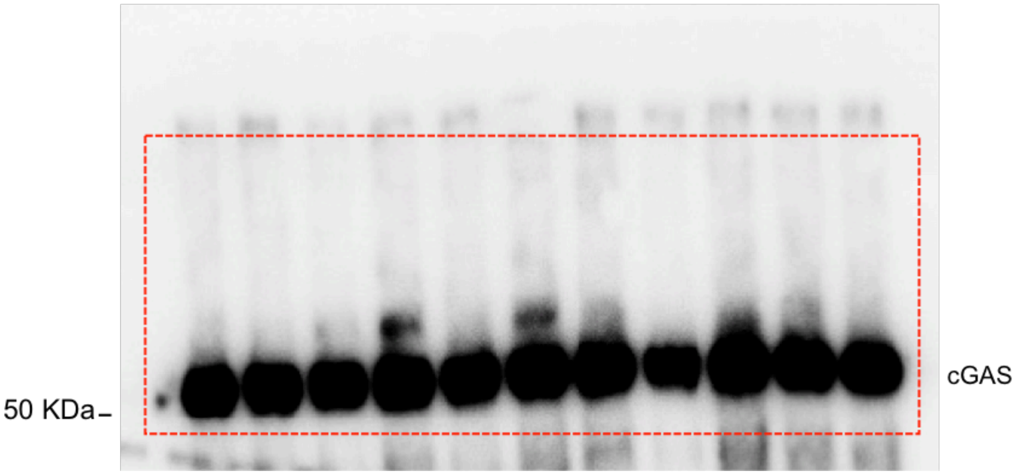

Supplementary Figure 10, cont. Full-length uncropped western blots.

Uncropped Western blot images for Supplementary Figure 4. Cropped areas are marked by red box.

Supplementary Table 1. Primers used for PCR

Gene Name	Direction	Primer sequence (5'-3')
Human TRIM56	Forward	GAGCTCGAGCTGTTTCCCACGGGTCCTCGCCCTCC
	Reverse	GATTCTAGAACTGTCCGGAGA ACG GAC CCG AAA
Mouse TRIM56 genomic PCR#1	Forward	AAGTGGAGGCAGGAAGTTCA
	Reverse	GACACGGTGCTTATGGGTCT
Mouse TRIM56 genomic PCR#2	Forward	CTACCTTTGGCCCTTGACCT
	Reverse	GGCCTTGACAAGATCCAAGA
Mouse TRIM56	Forward	GAAGAATTCGCCACCATGAACTCCAAAGACTCCTCCCCA
	Reverse	GAAACGCGTGCTGCAGGAAT GTTCCAAAGCAAGCAGTT
Mouse TRIM56 Mutant	Forward	AGCGATTTCCTAGCCT**C**TAAAATCT**C**CCTGGAGCAGTTA
	Reverse	TAACTGCTCCAGGGAGATTTTAGAGGCTAGGAAATCGCT
Mouse cGAS	Forward	GAGCTCGAGGCCACCATGGAAGATCCGCGTAGAAGGACGACG
	Reverse	GGTTCTAGAAAGCTTGTCAAAAATTGGAAA CCCATTATTTCT
McGASK278R	Forward	GAAGTTAAAGAAATCAGAGATATAGATGTCAGTGTG
	Reverse	CACACTGACATCTATATCTCTGATTTCTTTAACTTC
McGASK335R	Forward	GGCTGGCTGGGCACAAGAGTGAGGACCAATCTAAGA
	Reverse	TCTTAGATTGGTCCTCACTCTTGTGCCCAGCCAGCC
McGASK350R	Forward	TTTTATCTCGTA CC AGGAATGCAAAGGATGGAAAT
	Reverse	ATTTCCATCCTTTGCATTCCTGGGTACGAGATAAAA

Supplementary Table 2. shRNA or sgRNA sequence information

shRNA	Target sequence (5'-3')
Human TRIM56#1	GATTTCGAATGGGCAGTGA
Human TRIM56#2	AAGAGAATAGGCTACTGGA
Human TRIM56#3	CACCACCGCCGCTGCTATA
**sgRNA**	**Target sequence (5'-3')**
Mouse TRIM56#1	**CCA**GGACTGTCTGGCACAACTGG
Mouse TRIM56#2	AGTCCTGGCAATAGGTATGT**AGG**

Supplementary Table 3. Primers used for real-time PCR

Gene Name	Direction	Primer sequence (5’-3’)
Mouse IFN β	Forward	ATGAACTCCACCAGCAGA CAG
	Reverse	ACCACCATCCAGGCGTAGC
Mouse β-actin	Forward	GGCTATGCTCTCCCTCACG
	Reverse	CGCTCGGTCAGGATCTTCAT
Mouse IP-10	Forward	AAGTGCTGCCGTCATTTTCT
	Reverse	GTGGCAATGATCTCAACACG
Mouse TRIM56	Forward	ATGAACTCCAAAGACTCCTCCCCA
	Reverse	AGTTGGCTTCCCTGAATGGACGTCTCC
